# The Interactions between the Long Non-coding RNA NERDL and Its Target Gene Affect Wood Formation in *Populus tomentosa*

**DOI:** 10.3389/fpls.2017.01035

**Published:** 2017-06-15

**Authors:** Wan Shi, Mingyang Quan, Qingzhang Du, Deqiang Zhang

**Affiliations:** ^1^Beijing Advanced Innovation Center for Tree Breeding by Molecular Design, College of Biological Sciences and Technology, Beijing Forestry UniversityBeijing, China; ^2^National Engineering Laboratory for Tree Breeding, College of Biological Sciences and Technology, Beijing Forestry UniversityBeijing, China; ^3^Key Laboratory of Genetics and Breeding in Forest Trees and Ornamental Plants, Ministry of Education, College of Biological Sciences and Technology, Beijing Forestry UniversityBeijing, China

**Keywords:** association genetics, Nerdl, lncRna–mRna interactions, epistasis, wood formation

## Abstract

Long non-coding RNAs (lncRNAs) are important regulatory factors for plant growth and development, but little is known about the allelic interactions of lncRNAs with mRNA in perennial plants. Here, we analyzed the interaction of the NERD (Needed for RDR2-independent DNA methylation) *Populus tomentosa* gene *PtoNERD* with its putative regulator, the lncRNA NERDL (NERD-related lncRNA), which partially overlaps with the promoter region of this gene. Expression analysis in eight tissues showed a positive correlation between NERDL and *PtoNERD* (*r* = 0.62), suggesting that the interaction of NERDL with its putative target might be involved in wood formation. We conducted association mapping in a natural population of *P. tomentosa* (435 unrelated individuals) to evaluate genetic variation and the interaction of the lncRNA NERDL with *PtoNERD*. Using additive and dominant models, we identified 30 SNPs (*P* < 0.01) associated with five tree growth and wood property traits. Each SNP explained 3.90–8.57% of phenotypic variance, suggesting that NERDL and its putative target play a common role in wood formation. Epistasis analysis uncovered nine SNP-SNP association pairs between *NERDL* and *PtoNERD*, with an information gain of -7.55 to 2.16%, reflecting the strong interactions between NERDL and its putative target. This analysis provides a powerful method for deciphering the genetic interactions of lncRNAs with mRNA and dissecting the complex genetic network of quantitative traits in trees.

## Introduction

DNA methylation is a conserved epigenetic silencing mechanism that functions in many crucial plant growth and developmental processes, such as genomic imprinting and abiotic stress responses (Bird, 2002; Song et al., 2016). RdDM (RNA-directed DNA methylation), the most important and well-characterized DNA methylation pathway, is guided by 24-nt siRNAs, induces *de novo* cytosine methylation, and regulates gene expression (Le et al., 2014). RdDM occurs only in plants performing transcriptional gene silencing (TGS) involving the Pol IV–RDR2–DCL3 complex. Another siRNA-dependent DNA methylation pathway, the NERD (Needed for RDR2-independent DNA methylation)-dependent pathway (Matzke et al., 2015), involves RDR1/RDR6 and AGO2 and is guided by 21-nt siRNAs (Matzke and Mosher, 2014) to mediate gene silencing at the post-transcriptional level in *Arabidopsis thaliana*. The NERD pathway initiates *de novo* methylation of newly integrated transposons that initially undergo PTGS (post-transcriptional gene silencing), which may build a bridge between PTGS and TGS (Matzke and Mosher, 2014).

The NERD pathway requires Pol IV and Pol V, as well as several PTGS components, including NERD, RDR6, SGS3 (SUPPRESSOR OF GENE SILENCING 3), SDE3 (SILENCING DEFECTIVE 3), and SDE5 (Matzke and Mosher, 2014). NERD, a key protein in the NERD pathway, plays important roles in plant growth and development. In *Arabidopsis*, NERD is responsible for the accumulation of siRNA at NERD-targeted loci (Pontier et al., 2012). In rice, *OsNERD1* affects pollen sterility via its effect on related transposons, as well as cold-related transposons (Chen, 2015). Natural allelic variation plays an important role in plant development and physiology, especially plant growth and morphology, the timing of germination and flowering, and primary metabolism (Alonso-Blanco et al., 2009). However, the specific genetic variants resulting in variations in growth and development in woody plants are only starting to be uncovered. NERD was identified in *Populus trichocarpa* through database searches (Pontier et al., 2012), but the effect of allelic variations within NERD on tree growth and wood formation, which could be utilized for genetic improvement of important economic traits in forest trees, remains unknown.

LncRNAs (long non-coding RNAs), which are longer than 200 nucleotides and have no apparent coding ability, are emerging as important participants in numerous biological processes (Quan et al., 2015). In animals, lncRNAs are involved at multiple levels in many biological regulatory processes, interacting with their target genes via various mechanisms (Ponting et al., 2009). In humans, lncRNAs in the promoter regions of protein-coding genes are thought to affect the expression of these genes, thus playing an important role in controlling cell growth (Hung et al., 2011). Several plant lncRNAs have been identified and their roles characterized, such as lncRNA XLOC_057324 in panicle development and fertility in rice (Zhang et al., 2014), lncRNAs involved in flowering in *Arabidopsis* (Heo and Sung, 2011) and drought responses in *Populus* (Shuai et al., 2014). The effects of ncRNAs and their target genes on plant growth and development have been explored. For example, ASCO-lncRNA, which hijacks nuclear regulators of alternative splicing machinery by regulating the expression levels of its target gene, contributes to lateral root formation in *Arabidopsis* (Bardou et al., 2014). However, few studies have focused on the roles of lncRNAs in perennial trees.

Stem growth and wood formation are important processes in the lifecycles of perennial plants such as forest trees. However, the long cycle of forest tree growth, the presence of highly lignified tissues, and the lack of suitable mutants, have limited the use of traditional transgenic analysis techniques in trees to explore specific interactions between ncRNAs and their target genes. SNP-based association mapping in conjunction with molecular marker-assisted selection (Thumma et al., 2005) and association studies can be used to decipher the interactions of ncRNAs with mRNA and their roles in tree growth and wood formation. For instance, a SNP-based association analysis shed light on the involvement of the interactions between the miRNA *Pto-miR530a* and its target gene *Pto-KNAT1* in eight traits, which laid the foundation for exploring the multi-gene networks affecting tree growth and wood properties (Yang et al., 2015). However, the effects of the genetic interactions between lncRNAs and their targets on tree growth and wood formation remain largely unknown.

Here, we explored the genetic variation and interactions of the lncRNA NERDL and its putative target gene, *PtoNERD*, in a natural population of 435 unrelated individuals of the poplar *Populus tomentosa*, a major commercial tree species grown for pulp and timber production in northern China. Using SNP-based association mapping with additive and dominant models, we detected significant SNPs within *NERD* and *PtoNERD* associated with tree growth and wood properties, revealing the common role of NERDL and its potential target, *PtoNERD*, in wood formation. Expression profiling revealed a significant correlation between the expression of NERDL and *PtoNERD*, suggesting that they share a common pathway involved in wood formation. Epistasis analysis uncovered strong allelic genetic interactions between NERDL and *PtoNERD*. Our approach is useful for identifying genetic variation and deciphering the interactions between lncRNAs and mRNAs associated with multiple economic traits in forest trees.

## Materials and Methods

### Population Materials and Phenotypic Data

#### Plant Materials

The association population was composed of 435 unrelated individuals of *P. tomentosa* randomly selected from a collection of 1047 individuals covering the original distribution of *P. tomentosa*, as well as three climatic zones (southern, northwestern, and northeastern China) (Huang, 1992). A clonal arboretum of this collection was built in 1982 in Guan Xian County, Shandong Province, China (36°23′N, 115°47′E) using a randomized complete block design with three replications. Forty-four individuals were randomly selected from this association population to identify SNPs within *NERDL* and *PtoNERD* via direct sequencing.

#### Phenotypic Data

Phenotypic data for 10 growth and wood property traits were obtained from the 435 selected individuals in 2011 as previously described (Du et al., 2013b). Trees of this age are in a stable growth and developmental phase and can therefore be used to reflect the natural growth and developmental state of trees. Seven wood property traits and three tree growth traits were investigated: LC (lignin content, %), HC (holocellulose content, %), HEMC (hemicellulose content, %), AC (α-cellulose content, %), FL (fiber length, mm), FW (fiber width, μm), Angle (microfibril angle, °), H (tree height, m), D (tree diameter at breast, cm), and V (volume of wood, m^3^). The correlations of these phenotypic data in the association population were calculated as previously described (Du et al., 2013b).

### Identification of the lncRNA NERDL and Its Putative Target, *PtoNERD*

#### Isolation and Sequence Analysis of *PtoNERD*

Cambium, developing xylem, and mature xylem tissues were obtained from 1-year-old *P. tomentosa* clone LM50 when the tree growth and development were in a relatively active state, allowing us to obtain important information about tree growth and wood formation. Total RNA was extracted from the tissues with a Plant RNeasy Kit (Qiagen) following the manufacturer’s instructions. DNA contamination was removed with three additional on-column DNase digestions during RNA purification with an RNase-Free DNase Set (Qiagen). The three RNA samples were used to construct RNA-seq libraries, and transcript sequencing was performed on an Illumina HiSeq 2500 by Shanghai Biotechnology Corporation (Shanghai, China). High-quality reads were mapped to the *Populus* reference genome (Tuskan et al., 2006) using TopHat v2.0.9 (Trapnell et al., 2012). The transcripts were assembled using Cufflinks v2.1.1 (Trapnell et al., 2012), and the expression levels were calculated and normalized by FPKM (fragments per kilobase of transcript per million fragments). The sequencing data are available in the NCBI Sequence Read Archive (SRA accession number SRP073689). Expression analysis of seven genes (*RDR1, RDR6, NERD, SGS3, SDE3, SDE5*, and *AGO2*) involved in the NERD pathway showed that *PtoNERD* was highly expressed in vascular tissue, suggesting that it might function in wood formation (**Supplementary Figure S1**). Therefore, *PtoNERD* (Accession number: KY344444–KY344487) was selected as a candidate gene for further analysis. RNA sequencing was performed to identify the mRNA sequence of *PtoNERD*, which was compared with available *Arabidopsis* or *P. trichocarpa NERD* sequences, showing that *PtoNERD* mRNA shares high sequence similarity with *AtNERD* AT2G16485 (45%) and Potri.009G1238000 (95%).

To evaluate the phylogenetic relationship of *PtoNERD* with similar genes in other species, the amino acid sequences of NERD from *A*. *thaliana, P. trichocarpa, P*. *euphratica, Picea abies, Pinus taeda, Zea mays*, and *Oryza sativa* ssp. *japonica* were identified by searching public databases at NCBI^1^ using BLAST, and sequence alignment and phylogenetic tree construction were performed with MEGA 5.0 as previously described (Tamura et al., 2011).

#### Identification of an lncRNA Targeting *PtoNERD*

RNA sequencing was performed on lncRNAs from three tissues. All lncRNA sequences identified by transcriptome sequencing were aligned against the *Populus* reference genome (SRA accession number SRP073689) (Tuskan et al., 2006). To identify the lncRNA potentially targeting *PtoNERD*, genome annotation and a genome browser were used to screen potential *cis* lncRNAs that are physically close to *PtoNERD* (within 10 kb) as described (Jia et al., 2010).

### Tissue-Specific Expression Analysis of lncRNA and Its Putative Target, *PtoNERD*

Total RNA was extracted from fresh tissue samples from 1-year-old *P. tomentosa* clone LM50, including juvenile leaf, mature leaf, apical shoot meristem, phloem, cambium, developing xylem, mature xylem, and root tissue as described (Du et al., 2013a). The RNA was reverse-transcribed into cDNA using the SuperScript First-Strand Synthesis system with the supplied polythymine primers (Invitrogen, Life Technologies, New York, NY, United States) (Zhang et al., 2010a). All cDNA samples were used to investigate the tissue-specific expression of *PtoNERD* and NERDL through reverse-transcription quantitative PCR (RT-qPCR). The qPCR was performed on the 7500 Fast Real-Time PCR System with SYBR Premix EX Taq (TaKaRa, Dalian, China) with gene-specific primers (Supplementary Table S1) designed with Primer Express 3.0 (Applied Biosystems). The PCR conditions were as follows: 94°C for 5 min; 40 cycles of 94°C for 30 s, 58°C for 30 s, and 72°C for 30 s; and a final cycle at 70–95°C for melting curve analysis to confirm the specificity of the amplification. The qPCR was conducted as described by Du et al. (2013a). All reactions were conducted with three technical and three biological replicates. The results for the various tissues were standardized to the internal control (*Actin*, Accession number: EF145577). Data analysis was performed with Opticon Monitor Software 3.1 (MJ Research, Bio-Rad, Hercules, CA, United States).

### SNP Discovery and Genotyping

Total genomic DNA was extracted from fresh young leaves with a Plant DNeasy Mini kit (Qiagen China, Shanghai) according to the manufacturer’s instructions. The primer sets used for amplification and sequencing were specifically designed based on the sequence of the lncRNA NERDL and the cDNA sequence of *PtoNERD*. The 16,119 bp genomic DNA sequence of *PtoNERD*, including the flanking region (2000 bp), was obtained by direct sequencing of the *P. tomentosa* (LM50 clone) using conserved primers and the BigDye Terminator Cycle Sequencing kit version 3.1 (Applied Biosystems, Beijing, China) on a Li-Cor 4300 genetic analyzer (Li-Cor Biosciences, Lincoln, NE, United States). The Prosite database^2^ was used to identify protein domains, families, and functional sites, and SMART^3^ was used to analyze the protein sequence. To identify SNPs, *NERDL*, and *PtoNERD* were sequenced and analyzed in 44 unrelated individuals from the association population with MEGA 4.0 and DnaSP v4.90.1 (Rozas et al., 2003). Insertions/deletions (InDels) were disregarded in the analysis. All 88 sequences have been deposited in the GenBank database (GenBank Accession No. KY344444-KY344487 for *NERDL* and KY344488-KY344531 for *PtoNERD*). Sequence alignment was performed with BioEdit, and manual editing was performed to remove primer sequences and to confirm sequence quality. All common SNPs (minor allele frequencies > 0.05) detected in *NERDL* and *PtoNERD* were genotyped in the association population using the Beckman Coulter sequencing system (Franklin Lakes, NJ, United States).

### Nucleotide Diversity and Linkage Disequilibrium

#### Nucleotide Diversity

The summary statistics for SNP polymorphisms were obtained using DnaSP version 4.90.1 (Rozas et al., 2003). The average number of pairwise differences per site between the sequences, π (Nei, 1987), and the average number of segregating sites, 𝜃_w_ (Watterson, 1975) were used to assess the nucleotide diversity. Diversity statistics were also calculated for non-coding, synonymous, and non-synonymous sites. Tajima’s D and Fu and Li’s D (Tajima, 1989; Fu and Li, 1993) statistics were obtained and the complete data set was tested using 10,000 simulations to determine whether a gene or genomic region is evolving randomly (neutral evolution) or whether the region is under selection (non-neutral evolution). The statistical significance of Tajima’s D statistics was determined using DnaSP v4.90.1.

#### Linkage Disequilibrium (LD)

Linkage disequilibrium between pairs of SNP markers within *NERDL* and *PtoNERD* were assessed using TASSEL V.5.0 (Bradbury et al., 2007) as the squared correlation of allele frequencies (*r*^2^), which can be affected by both recombination and differences in allele frequencies between sites (Hill and Robertson, 1968). To assess the level of LD within the sequenced regions of *NERDL* and *PtoNERD*, the decay of LD with featured physical distance (bp) between common SNP sites within both candidate genes was estimated by non-linear regression analysis with 10^5^ permutations of the data (Remington et al., 2001), and singletons were excluded.

### Detection of SNP-Based Phenotype–Genotype Associations

#### Single SNP-Based Associations

The phenotypic trait–SNP genotype associations between 159 common SNP markers within *NERDL* and *PtoNERD* and 10 traits were identified using a MLM (mixed linear model) with 10^5^ permutations with the software package TASSEL v5.0^4^ (Yu et al., 2006). The values of estimated membership probability (*Q*) and pairwise kinship (*K*) were used to evaluate the effects of population structure and relatedness among individuals for marker–trait associations. In this *Q* + *K* model, *Q* matrix was obtained based on the significant subpopulations (*k* = 3) (Du et al., 2012) using STRUCTURE version 2.3.4^5^ (Pritchard et al., 2000), and K, the relative kinship matrix, was evaluated with SPAGeDi version 1.3 (Hardy and Vekemans, 2002) using the method proposed by Ritland (1996). The positive FDR method was used to correct for multiple testing using QVALUE software (Storey and Tibshirani, 2003). Additive effects and dominance effects were calculated with Tassel v5.0 to analyze the patterns of gene action.

#### Haplotype Analysis

Haplotype frequencies were calculated with SNP genotype data, and haplotype association tests were performed on a three-marker sliding window using haplotype trend regression software (Higuchi, 2012). The significance of each haplotype association was based on 10^4^ permutation tests, and singleton alleles and haplotypes with frequencies less than 0.05 were excluded from further analysis.

#### Multi-SNP-Based Epistasis Analysis

To detect the epistatic effects among multiple factors, a multifactor dimensionality reduction (MDR) algorithm (Hahn et al., 2003) was developed. With this algorithm, it is possible to process high-dimensionality genetic data into a single dimension, allowing interactions to be investigated using a relatively small set of data. MDR 3.0.2, the Relief algorithm, was used to filter all unlinked SNPs (*r*^2^ < 0.1 or different genes), improving the reliability of probability approximation. The five most-significant SNPs were calculated for each trait. The genetic effects of significant SNP–SNP pairs were assessed by IG (information gain) analysis, which was calculated via an entropy-based measurement (Moore et al., 2006).

### Transcript Analysis of SNP Genotypes

The overlapping significant SNPs were used to investigate the roles of different genotypes on the expression of NERDL and *PtoNERD*. For each SNP genotypic class, 10 individuals were selected and used to measure the expression of the lncRNA NERDL and *PtoNERD* via RT-qPCR. The differential expression across different genotypes was calculated with ANOVA. The effects of different genotypes of the shared locus on phenotypic variation were also investigated.

## Results

### Sequence Analysis of *NERDL* and Its Putative Target *PtoNERD* from *P. tomentosa*

**FIGURE 1:**
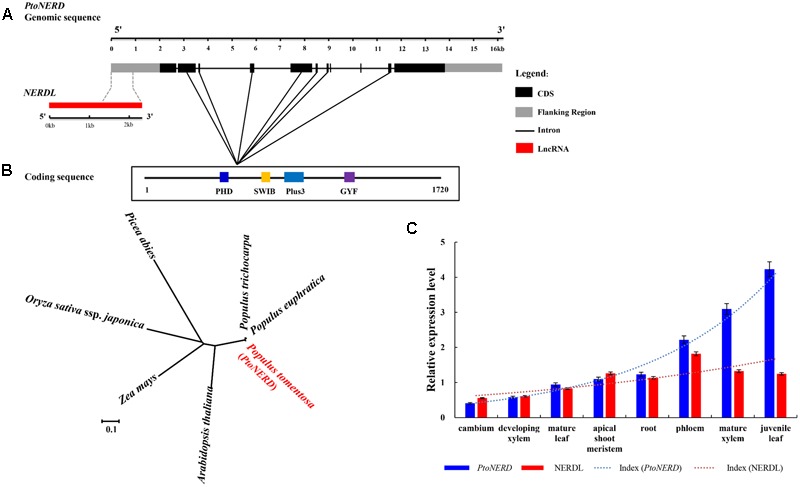
Gene structures of *NERDL* and *PtoNERD*, expression levels of NERDL and *PtoNERD*, and phylogenetic tree of *PtoNERD.* **(A)** Gene structures and SNP diversity of *NERDL* and *PtoNERD.* NERDL shares a 960 bp long overlapping region with *PtoNERD*. **(B)** Phylogenetic tree of homologs of *Arabidopsis thaliana, P. trichocarpa, Populus euphratica, Picea abies, Pinus taeda, Zea mays*, and *Oryza sativa* ssp. *Japonica.* **(C)** The relative expression levels of NERDL and *PtoNERD* in eight tissues measured by RT-qPCR with *Actin* as the internal control.

To identify SNPs within *PtoNERD*, we cloned a cDNA from *PtoNERD*. This cDNA is 9527 bp long, including a 5163 bp open reading frame and 364 bp of 3′UTR. Based on the alignment of the full-length cDNA sequence to the genomic sequence, *PtoNERD* (16119 bp) has 10 introns and 11 exons flanked by 2000 bp of promoter region, 364 bp of 3′UTR, with a 2000 bp flanking region (**Figure 1A**); the length of each region is listed in **Table 1**. *PtoNERD* encodes a putative polypeptide of 1720 amino acids, with an estimated molecular mass of 188.4 kD and a pI of 4.82. Characterization of the amino acid sequence of *PtoNERD* revealed that it contains the essential domain PHD, SWIB, Plus-3, and GYF (**Figure 1A**). Phylogenetic analysis indicated that *PtoNERD* is an ortholog of *AtNERD* and *PtrNERD* (**Figure 1B**). Phylogenetic analysis using the neighbor-joining method showed that *PtoNERD* in *P. tomentosa* shares a close relationship with the homologous sequences in *P. trichocarpa* and *P. euphratica*, indicating that *PtoNERD* is highly conserved in *Populus*, whereas the conservation of *PtoNERD* in distantly related species is weak.

**Table 1:** Single nucleotide polymorphisms of *NERDL* and *PtoNERD*.

Gene	Region	Length (bp)	Number of polymorphic sites	Frequency (bp^-1^)	Nucleotide diversity	
					π	𝜃w
*NERDL*	*NERDL*	2286	26	88	0.00257	0.00261
*PtoNERD*	Promoter	2000	28	71	0.0032	0.00322
	Exon 1	669	0	0	0	0
	Intron 1	63	0	0	0	0
	Exon 2	728	1	728	0.00028	0.00032
	Intron 2	120	0	0	0	0
	Exon 3	80	0	0	0	0
	Intron 3	2,051	5	410	0.00055	0.00056
	Exon 4	188	0	0	0	0
	Intron 4	1459	27	54	0.00402	0.00425
	Exon 5	931	3	310	0.00058	0.00074
	Intron 5	104	0	0	0	0
	Exon 6	128	0	0	0	0
	Intron 6	344	0	0	0	0
	Exon 7	60	0	0	0	0
	Intron 7	74	0	0	0	0
	Exon 8	51	0	0	0	0
	Intron 8	1202	13	92	0.0023	0.00249
	Exon 9	71	0	0	0	0
	Intron 9	1076	10	108	0.00139	0.00214
	Exon 10	155	1	155	0.00084	0.00148
	Intron 10	99	1	99	0.00131	0.00232
	Exon 11	2102	19	111	0.00161	0.00208
	3′UTR	364	3	121	0.00139	0.00189
	Flanking region	2000	31	65	0.00276	0.00356
	Total silent^a^	12104.46	125	97	0.00206	0.00237
	Synonymous	1146.46	7	164	0.00099	0.0014
	Non-synonymous	4013.54	17	236	0.00078	0.00097
	Total^b^	16119	142	114	0.00174	0.00203

*^a^Total silent: synonymous sites plus polymorphic sites in non-coding regions of PtoNERD.*

*^b^Total: silent sites plus non-synonymous sites of PtoNERD.*

To further explore the roles of *PtoNERD* in plant development, using genome annotation and a genome browser, we identified *NERDL*, an lncRNA located in the antisense strand and overlapping with the promoter region of *PtoNERD*, which acts as a *cis*-regulator of *PtoNERD*. Sequencing analysis revealed the 2286-nt sequence of *NERDL*, which shares an overlapping region of 961 bp with *PtoNERD* (**Figure 1A**). Therefore, *PtoNERD* was regarded as the candidate *cis* target gene of the overlapping lncRNA NERDL (lncRNA related to *PtoNERD*). Several functional motifs are found in the overlapping region, such as light responsive element 3-AF1 binding site, Box I, and GT1-motif, as well as as-2-box involved in shoot-specific expression and light responsiveness.

### *NERDL* and *PtoNERD* Expression Are Highly Correlated

We explored the tissue-specific expression patterns of the lncRNA NERDL and *PtoNERD* in eight different plant tissues via RT-qPCR (**Figure 1C**). NERDL was highly abundant in phloem (1.82 ± 0.09) (arbitrary units normalized to control), followed by mature xylem (1.33 ± 0.07), with the lowest abundance detected in cambium (0.56 ± 0.03), suggesting that NERDL plays a regulatory role in wood formation. *PtoNERD* mRNA was enriched in juvenile leaves (4.23 ± 0.20), followed by mature xylem (3.10 ± 0.14), with the lowest abundance in cambium (0.41 ± 0.19). Overall, *PtoNERD* was more highly expressed than NERDL. The Pearson’s product moment correlation was 0.62 (*P* < 0.05), indicating a strong correlation in transcript levels between NERDL and *PtoNERD*. The reads numbers of NERDL and *PtoNERD* obtained by RNA-seq showed a similar trend in cambium, developing xylem, and mature xylem tissue (**Supplementary Figure S3**).

### Single Nucleotide Diversity and Linkage Disequilibrium in *NERDL* and Its Putative Target *PtoNERD*

To characterize the intraspecific polymorphisms for use in association mapping, we obtained the full-length genomic sequences of *NERDL* and *PtoNERD* from 44 unrelated individuals of *P. tomentosa*. In total, 26 SNPs were detected in *NERDL* at a density of 1/88, and 142 SNPs in *PtoNERD* were detected at a frequency of approximately one SNP every 114 bp. The nucleotide diversity (excluding InDels) was higher in *NERDL* (π = 0.00257 and 𝜃w = 0.00261) than in *PtoNERD* (π = 0.00174 and 𝜃w = 0.00203). As expected, the average nucleotide diversity in *PtoNERD* was higher in introns (π = 0.00096) than in exons (π = 0.00030), since under selection pressure, the coding region of a gene shows more conservation than the non-coding regions (**Table 1**). Within the coding region of *PtoNERD*, the average nucleotide diversity of synonymous polymorphisms (*d*_S_, π = 0.00099) was higher than that of non-synonymous polymorphisms (*d*_N_, π = 0.00078) at a ratio of *d*_N_/*d*_S_ (0.788 < 1), indicating that non-synonymous sites in the exons are under purifying selection.

To perform LD analysis, 24 and 135 common SNPs (minor allele frequency > 0.05) from *NERDL* and *PtoNERD*, respectively, were successfully genotyped across 435 individuals in the association population. Four common SNPs were located in the overlapping region and were shared by *NERDL* and *PtoNERD* (SNP21–24 in *NERDL* and SNP1–4 in *PtoNERD*). A plot of the *r*^2^ combined with physical distance between SNPs within *NERDL* and the *PtoNERD* genes is shown in **Supplementary Figure S2**. In this population, the rate of LD declined rapidly, and the *r*^2^ values decreased to 0.1 within 1200 and 8000 bp in *NERDL* (**Supplementary Figure S2A**) and *PtoNERD* (**Supplementary Figure S2B**), respectively, indicating that LD does not extend across the entire gene region.

### Genetic Effect of Allelic Variation in *NERDL* and Its Target Gene Revealed by Association Studies

#### Single SNP-Based Association

To thoroughly investigate the effect of NERDL and its putative target *PtoNERD* on growth and wood formation in *P. tomentosa*, we performed an association study among 159 common SNPs from *NERDL* and *PtoNERD* and 10 quantitative traits using MLM. In all, we detected 35 significant associations (*P* < 0.01, *Q* < 0.10) between 30 unique SNPs and 5 quantitative traits (Supplementary Table S2), with the phenotype variance (*r*^2^) explained by each association ranging from 3.90 to 8.57%. Four SNPs in *NERDL* are associated with three growth and wood property traits in *P. tomentosa* (D, AC, and HEC), with an average *r*^2^ of 5.78%. For *PtoNERD*, 26 significant SNPs from the promoter, exon, intron, and 3′UTR regions are associated with five traits (D, AC, HEC, FW, and LC), with *r*^2^ ranging from 3.90 to 8.57%. Of the seven SNPs in the coding region, three non-synonymous SNPs (SNP33, SNP49, and SNP75) were detected that are significantly associated with FW (*r*^2^, 4.13–7.76%). For example, SNP33, a missense mutation in exon 1, results in an encoded amino acid change from Thr to Ile, which is significantly associated with FW.

Among the identified associations, the most-significant SNP–trait associations affected wood property traits, including 14 for FW and 9 for wood chemical composition (AC, HEC, and LC); of these associations, 11.54% involve SNPs within *NERDL*. In addition, three traits (D, AC, and HEC) formed 1–14 associations with SNPs from both *NERDL* and *PtoNERD* simultaneously, with various contributions to phenotypic variation, suggesting that NERDL and its target gene *PtoNERD* play common roles in phenotypic variation (Supplementary Table S2). These finding, combined with the high correlation between NERDL and *PtoNERD*, suggest that NERDL and its target may play a common role in wood formation. For example, two SNPs in *NERDL* and seven in *PtoNERD* were associated with D, with *r^2^* ranging from 4.21 to 6.10%. In addition, SNP9 from *NERDL* and four SNPs (SNP9, SNP11, SNP12, and SNP105) in *PtoNERD* were associated with more than one trait. Interestingly, an overlapping significant SNP locus, L23/N3, was detected in *NERDL* (L23, short for SNP23) and its putative target gene *PtoNERD* (N3, short for SNP3) that associated with D (*r*^2^ = 4.70%). In general, the single SNP-based associations of lncRNA NERDL and its potential target suggest that they play a role in tree growth and wood properties.

**FIGURE 2:**
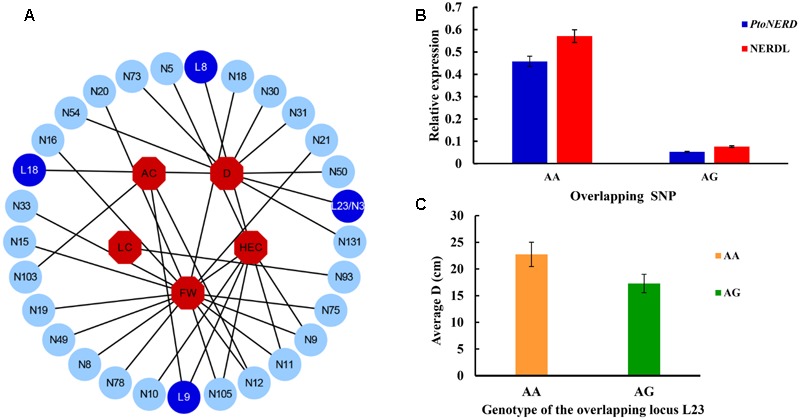
SNP-trait associations under additive and dominant effects in the association population of *P. tomentosa*, expression levels of genotypic classes for the overlapping SNP loci, and variations in average values of D depending on the genotype of L23/N3. **(A)** The associations with additive and dominant effect between 30 SNPs from *NERDL* and its target and five traits. Dark blue and light blue circles represent SNPs from *NERDL* (L) and *PtoNERD* (N), respectively, and red octagons indicate the five associated traits. **(B)** Expression levels of genotypic classes for the overlapping SNP loci. Error bars indicate standard deviations. **(C)** Average D depends on the genotype (AA or AG) of L23/N3.

To further examine the allelic roles of NERD and *PtoNERD* in tree growth and wood formation, we investigated the additive and dominant effects of the 35 SNP–trait associations, with an average *r*^2^ of 5.48% (**Figure 2A**). The additive effect of these associations varied from 0.17 to 14.50% (**Figure 2A** and **Table 2**). In particular, SNP12 in *PtoNERD*, associated with AC, had the largest additive effect (14.50%); this additive effect was lower when associated with FW (2.80%) (Supplementary Table S2). The dominant values of these associations ranged from -10.34 to 12.32%, with 68.57% possessing positive dominant effects and the trait FW having the most associations. SNP12 in *PtoNERD* associated with AC and had the largest positive dominant effect, but it had a negative dominant effect when associated with FW. SNP 8 in *NERDL* had the largest negative dominant effect (Supplementary Table S2).

**Table 2:** Summary of additive and dominant effects of all significant SNPs associated with each trait in the association population of *Populus tomentosa*.

Associated traits	Number of SNPs		Range of additive effect (%)	Number of positive or negative effects		Range of dominant effect (%)	Range of *R*^2^ (%)
	*NERDL*	*PtoNERD*		Positive	Negative		
**Growth traits**						
D (cm)	2	7	0.8∼6.45	7	2	-9.98∼7.42	4.21∼6.10
**Wood property traits**						
FW (μm)	0	14	0.22∼3.42	10	4	-2.30∼2.74	3.90∼8.57
AC (%)	1	2	7.85∼14.50	3	0	6.80∼12.32	5.64∼6.88
HEC (%)	2	6	2.1∼12.95	5	3	-10.34∼11.77	4.10∼6.54
LC (%)	0	1	0.17	0	1	-0.05	4.46

*D, diameter at breast height; FW, fiber width; AC, α-cellulose content; HEC, hemicellulose content; LC, lignin content.*

To further explore whether the allelic variation in the overlapping SNP L23/N3 influences the expression of lncRNA and its putative target, we used RT-qPCR to measure the transcript levels of NERDL and *PtoNERD*. The presence of different genotypes of L23/N3 led to significant differences in the transcript levels of NERDL and *PtoNERD* (**Figure 2B**). The highest *PtoNERD* mRNA levels were detected in the AA genotype (0.46 ± 0.02), followed by the AG genotype (0.05 ± 0.002). For NERDL, the expression levels were higher for the AA genotype (0.57 ± 0.02) than for the AG genotype (0.08 ± 0.004), indicating that the same locus can influence the expression of different transcripts. In addition, the average D depended on the genotype of L23/N3, with an average D of 22.74 cm in this association population for the AA genotype and 17.28 cm for AG (**Figure 2C**).

#### Haplotype-Based Associations

**FIGURE 3:**
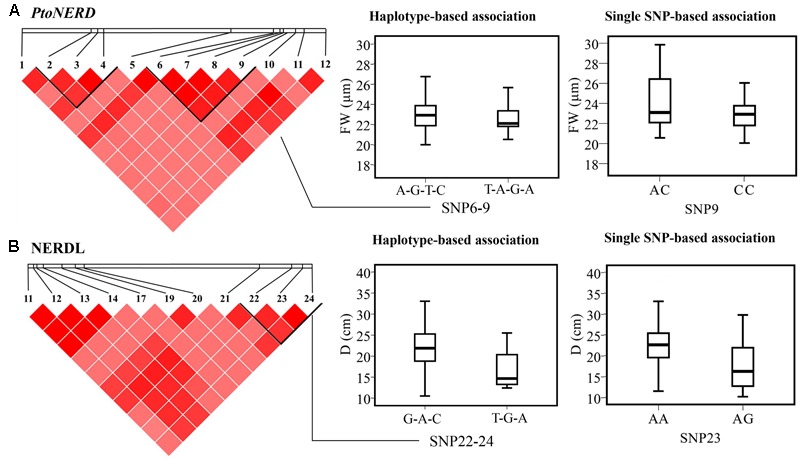
Haplotype-based epistasis associations. **(A,B)** The genotypic effect for significant haplotypes of NERDL*_*SNP22-24 and *PtoNERD_*SNP6-9 with that of the single loci of NERDL*_*SNP23 and *PtoNERD_*SNP9 based on single SNP-based associations.

We detected 15 common haplotypes (frequency ≥ 0.05) within *NERDL* (3) and *PtoNERD* (14) associated with four traits (D, FW, AC, and HEC), explaining 1.06–13.16% of the phenotypic variance (**Table 3**). Two haplotypes (G-A-C and T-G-A from NERDL_SNP22-24/PtoNERD_SNP2-4) associated with D were shared by *NERDL* and *PtoNERD* since they share an overlapping region. Each of these four traits is associated with one to nine haplotypes. For example, FW is associated with nine haplotypes from *PtoNERD* (*r*^2^, 1.06–2.85%) (**Table 3**). In addition, one haplotype (T-A-G-A) from *PtoNERD*_SNP6-9 is associated with both HEC and FW, with different *r*^2^ (2.08 and 2.72%, respectively), implying the pleiotropy effects and conspicuousness of the gene. In total, the results of haplotype association analysis were validated by single-SNP-based association analysis and explained more phenotypic variation. In addition, the association of L23/N3 with D supports the notion that the two overlapping haplotypes (G-A-C and T-G-A) from *NERDL* and *PtoNERD* are associated with the same trait, D, indicating that different SNPs located in different regions might have a common effect on the same trait (**Figures 3A,B**).

**Table 3:** Significant haplotypes from *NERDL* and its putative target *PtoNERD* associated with growth and wood properties traits in the association population of *Populus tomentosa*.

Traits	Significant haplotypes	Position	*P*-value	*Q*-value	Haplotype frequency	*R*^2^ (%)	Single-marker associations
**D (cm)**					
	NERDL_SNP22-24/*PtoNERD*_SNP2-4	Promoter				8.61	L23/N3 (D, Q = 0.06008)
	G-A-C		2.33E-02	6.98E-02	9.02E-01		
	T-G-A		3.76E-02	7.59E-02	5.16E-02		
	*PtoNERD*_SNP30-31	EXON1				2.88	SNP30 (D, Q = 0.0783)
	T-A		7.80E-03	6.60E-02	8.97E-01		SNP31 (D, Q = 0.07833)
	A-C		4.61E-02	7.86E-02	8.62E-02		
**FW (μm)**					
	*PtoNERD*_SNP6-9	Promoter				2.72	SNP9 (FW & HEC, Q = 0.0713)
	A-G-T-C		4.69E-02	7.92E-02	8.62E-01		
	T-A-G-A		7.01E-02	8.05E-02	1.03E-01		
	*PtoNERD*_SNP13-17	Promoter				1.22	SNP15 (FW, Q = 0.0769)
	A-C-C-C-G		7.72E-02	8.67E-02	8.91E-01		
	*PtoNERD*_SNP18-19	Promoter				1.06	SNP18 (FW, Q = 0.0775) SNP19 (FW, Q = 0.0780)
	G-C		3.71E-02	7.34E-02	9.08E-01		
	T-T		3.71E-02	7.35E-02	9.20E-02		
	*PtoNERD*_SNP20-21	Promoter				1.63	SNP20 (FW, Q = 0.0781) SNP21 (FW, Q = 0.0781)
	G-G		2.85E-02	7.18E-02	8.74E-01		
	A-T		3.04E-02	7.25E-02	1.21E-01		
	*PtoNERD*_SNP44-49	EXON2				2.85	SNP49 (FW, Q = 0.0786)
	C-A-G-G-G-C		7.40E-02	8.12E-02	8.51E-01		
	T-C-A-C-A-G		7.92E-02	8.54E-02	9.77E-02		
**AC (%)**					
	NERDL_SNP7-10	–				13.16	SNP9 (AC, Q = 0.0678)
	T-C-T-C		1.08E-02	6.78E-02	1.09E-01		
**HEC (%)**					
	*PtoNERD*_SNP6-9	Promoter				2.08	SNP8 (HEC, Q = 0.0692) SNP9 (HEC, Q = 0.0703)
	T-A-G-A		3.53E-02	7.29E-02	1.03E-01		

*L23 for SNP23 in *NERDL* and N3 for SNP3 in *PtoNERD*.*

#### Epistatic Modeling Demonstrates the Strong Genetic Interactions of lncRNA NERDL with Its Target

We uncovered strong interactions of different genes with phenotypic variation by investigating the epistatic effects between the lncRNA NERDL and its target. In total, we detected 90 significant SNP pairwise associations (*P* < 0.01, *Q* < 0.1) representing 32 SNPs from *NERDL* (2 SNPs) and *PtoNERD* (30 SNPs) (Supplementary Table S3), including nine SNP–SNP pairs representing lncRNA–mRNA interactions (**Table 4**). The effects of these nine lncRNA–mRNA interactions ranged from -7.55 to 2.16%, and nearly half showed negative IGs, indicating the redundant information for the same traits.

**Table 4:** The SNP pairs and their main effects detected from *NERDL* and *PtoNERD* under the epistasis model in the association population of *Populus tomentosa*.

Associated traits	Attribute (*NERDL*)	Single effect (% *NERDL*)	Attribute (*PtoNERD*)	Single effect (% *PtoNERD*)	Effect of interaction (%)	Information gain (%)
**D (cm)**					
	L20	0.0379	N72	1.19	7.14	2.16
	L20	0.0379	N80	1.45	3.48	-1.77
	L20	0.0379	N81	1.14	2.12	-2.81
	L20	0.0379	N135	12.28	8.52	-7.55
**FW (μm)**					
	L3	0.0011	N11	2.48	1.53	-1.05
	L3	0.0011	N12	4.59	2.96	-1.73
	L3	0.0011	N20	2.13	2.77	0.53
	L3	0.0011	N98	0.28	0.97	0.58
	L3	0.0011	N135	0.69	1.39	0.59

**FIGURE 4:**
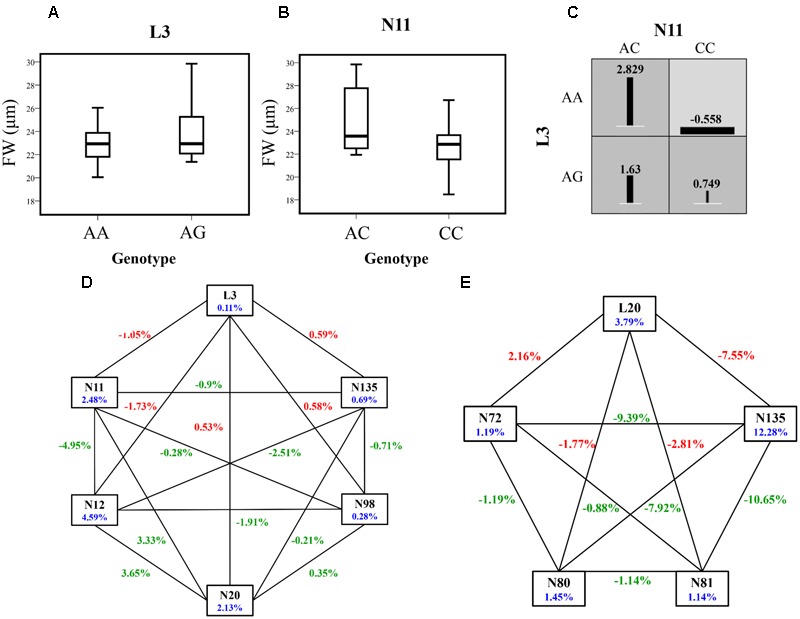
The epistatic network within the SNPs from *NERDL* and its target gene *PtoNERD*, and the phenotypic variations of pairwise genotypic combinations and single-locus genotypes. **(A,B)** Box plots showing the single-locus phenotypic variation of different genotypes of SNPs. **(C)** Square boxes show the pairwise phenotypic variation in different genotypic combinations of SNP pair L3-N11. **(D,E)** Interaction graph for FW and D among SNPs in *NERDL* and *PtoNERD*. Blue numbers represent the single-marker effect, red numbers indicate the pairwise epistatic effect between SNPs from *NERDL* and *PtoNERD*, respectively, and green numbers indicate the pairwise epistatic effect between SNPs from *PtoNERD*.

Of these associated SNPs detected in the epistasis model, 23.08% of unique SNPs also showed significant associations under the additive and dominant models (**Tables 2, 4**). The epistasis network consisting of SNPs from *NERDL* and its putative target offered strong genetic evidence for the lncRNA–mRNA interactions and their potential function in wood formation and tree growth (**Figures 4D,E**). For example, L3 and N11 showed an epistatic interaction for FW, and the epistasis value for the combination of the AA genotype of L3 and the AC genotype of N11 was higher than those of the other combinations. In addition, the epistasis values of the AA-AC combination (2.829%) in SNP pair L3-N11 were significantly higher than the values for single genotypes (-0.201% for AA and 2.229% for AC) (**Table 4**, Supplementary Table S3, and **Figures 4D,E**). L3 formed five SNP pairs with SNPs from the target gene, showing different pairwise effects (0.97–2.96%) on FW, indicating the pleiotropy of L3 and the important roles of these interactions in tree growth and development.

## Discussion

### Characterization of the lncRNA NERDL and Its Putative Target Gene PtoNERD

Unlike microRNAs, whose roles in post-transcriptional gene regulation are well-characterized (Bartel, 2009), the biological functions of lncRNAs are still largely unknown, although studies have increasingly focusing on the functional mechanisms of lncRNAs. LncRNAs function in a variety of process in plants, such as flowering, RdDM, and responses to biotic and abiotic stress (Quan et al., 2015). LncRNAs function through various mechanisms, including mutual interactions with RNAs and proteins (Guhaniyogi and Brewer, 2001). Base pairing-dependent lncRNA–mRNA interactions are a common mode of action for such interactions. In this study, we found that the lncRNA NERDL partly overlaps with the promoter region of *PtoNERD* and shares a 961 bp fragment containing four common SNPs and two haplotypes (**Figure 1A** and **Tables 2, 3**), suggesting a potential correlation between this lncRNA and *PtoNERD*. The overlap of lncRNAs with the promoter regions of protein-coding genes can affect the expression of these genes (Hung et al., 2011). Transcript expression analysis revealed a Pearson Correlation Coefficient of 0.62 (*P* < 0.05), providing evidence for a strong correlation between NERDL and its putative target gene, *PtoNERD* (**Figure 1C**). LncRNAs are usually shorter and are expressed at lower levels than protein-coding transcripts in *P. tomentosa* (Chen et al., 2015) and in mammals (Pauli et al., 2012). Here, RT-qPCR analysis and RNA-seq showed that the expression level of *PtoNERD* was higher than that of NERDL and that they both exhibit tissue-specific expression in *P. tomentosa* (**Figure 1C** and **Supplementary Figure S3**). NERDL was expressed at the highest level in phloem, suggesting that it may play a regulatory role in secondary wood formation. Furthermore, NERDL and *PtoNERD* were highly expressed in xylem (**Figure 1C**), suggesting that they play important roles in wood formation.

To understand the forces of evolutionary change and to evaluate the precision and power of association mapping, thorough knowledge of the levels of nucleotide diversity and the extent of LD in natural populations is required (Ingvarsson, 2008; Zhang et al., 2010b). In general, we found that the levels of average nucleotide diversity were lower in coding regions than in the non-coding regions (**Table 1**), implying that the coding regions are conserved relative to the other regions under natural pressure, with a dN/dS ratio (0.7879) less than 1 for *PtoNERD* (Neale and Kremer, 2011). Indeed, synonymous mutations occurring during evolution are more likely to be fixed than neutral ones owing to purifying selection (Zhang et al., 2010b). Also, the nucleotide diversity of *NERDL* (π *=* 0.00257) is slightly higher than that of *PtoNERD* (π *=* 0.00174), further confirming the conservative nature of the protein-coding gene. SNP diversity in genes has primarily been explored in exons, introns, and UTRs, while less attention has been paid to promoter regions and lncRNAs. It would be quite useful to analyze the levels of nucleotide diversity within the promoters of numerous genes compared with other gene regions. In the current study, we found that the promoter region possessed a significantly higher frequency of SNPs than the other regions in *PtoNERD* (π = 0.0032; **Table 1**). This finding suggests that this region may be an unstable regulatory region that plays important roles during the evolutionary process (Weickert et al., 2012).

### The Association of SNPs in *NERDL* and *PtoNERD* with Tree Growth and Wood Property Traits

We identified 4 significant SNPs in *NERDL* and 26 in *PtoNERD* associated with D, FW, AC, HEC, and/or LC, and 15 haplotype-based associations that were strongly supported by single-marker associations (**Table 3**), leading to the suggestion that NERDL and its potential target might play a role in tree growth and wood formation. We identified 35 associations with both additive and dominant effects associated with five traits (D, FW, AC, HEC, and LC), representing 30 significant SNPs from *NERDL* and *PtoNERD* (**Figure 2A** and **Table 2**), suggesting that the lncRNA–mRNA pair might have a common effect on certain traits. Eight SNPs from *NERDL* and *PtoNERD* were associated with HEC, with additive and dominant effects (*r*^2^, 4.10–6.54%), an association considered to indicate generalized pleiotropy (Mackay et al., 2009). Potential pleiotropy within trait categories and within different phenotypic traits has been reported in *P. trichocarpa* (Porth et al., 2014). Together, these findings suggest that NERDL and its putative target *PtoNERD* might affect HEC through a shared pathway. A similar trend was found for D, which is associated with nine SNPs from *NERDL* and *PtoNERD* (*r*^2^, 4.21–6.10%). Considering the associations based on additive and dominant effects, our findings suggest that NERDL and *PtoNERD* co-regulate HEC and D and that the role of NERDL might be similar to that of its target, *PtoNERD*. Furthermore, under the additive and dominant models, several SNPs are associated with multiple traits but with different contributions, such as SNP9 in *NERDL*, which is associated with two traits, with *r*^2^ varying from 11.28 to 13.08%, revealing the pleiotropy of specific SNPs, as observed in a previous study (Yang et al., 2015).

Three non-synonymous markers (SNP33, SNP49, and SNP75), one synonymous marker (SNP105), and 10 non-coding markers in promoter and introns were associated with FW simultaneously (Supplementary Table S2), confirming the necessity of using an integrated approach to characterize the genetic basis of wood traits. In addition, the existence of seven markers in exons suggests that these differences in *PtoNERD* might affect phenotypic variation by causing amino acid changes or influencing gene expression levels, whereas the five SNPs in introns might affect the regulatory roles of introns or exon splicing, thereby leading to phenotypic variation. One common SNP (L23/N3) overlapping in *NERDL* and *PtoNERD* was significantly associated with D (*r*^2^ = 4.7%). Notably, genotypes of the overlapping SNP L23/N3 led to significant differences in the transcript levels of NERDL and *PtoNER*D (**Figure 2B**). The transcript levels of the AA and AG genotypes showed similar trends in both NERDL and its putative target *PtoNERD*, indicating that this overlapping locus can influence the expression of different transcripts. Overall, the average D was higher in the AA genotype (22.74 cm) than in the AG genotype (17.28 cm) (**Figure 2C**), indicating that variations in genotype may lead to changes in phenotype. Moreover, two shared common haplotypes from NERDL_SNP22-24/*PtoNERD*_SNP2-4 associated with D surround L23/N3. The locus might serve as a functional marker involved in the regulation of D, which should be explored in the future.

### The Interactions of the lncRNA NERDL and Its Potential Targets Have Epistatic Effects on Wood Formation

Epistasis analysis is a crucial method for deciphering the effects of genetic variation and interactions (Mackay, 2014). Epistasis analysis can address the limitations of single-locus analysis and is a convenient method for further investigating the interactions of lncRNAs and mRNA and their effects on quantitative traits (Quan et al., 2016). Complex gene interactions have a stronger effect on disease susceptibility than single genes (Chou et al., 2011). Here, we characterized nine significant lncRNA–mRNA associations involved in two traits (D and FW) and 11 unique SNPs from *NERDL* and *PtoNERD* (**Figures 4D,E**), reflecting the possible genetic interaction of the lncRNA NERDL with *PtoNERD*. Furthermore, 55.56% of these SNP–SNP pairs had negative IG values, implying that redundant information is carried in the pairs for each trait and that their similar functional modes contribute to phenotypic variation. In associations with positive IG values, the contribution of pairwise interactions to phenotypic variation is more than the sum of the contributions of two SNP individuals. The genotypic combinations with the highest and lowest IG values for FW were found for SNP pair L3-N11 (**Figures 4A–C**), where the value for the genotypic combinations was significantly higher than that of the single-locus genotype. The identification of positive and negative IGs contributes to our understanding of the close interactions of NERDL with its target.

Of the SNPs showing lncRNA–mRNA interactions identified under the epistasis model, L3 in *NERDL* formed five SNP pairs with N11, N12, N20, N98, and N135 in *PtoNERD* under the epistasis model associated with FW with diverse IGs, revealing the complexity of the interactions between NERDL and *PtoNERD* (**Figure 2B**). L20 also showed similar associations with four SNPs associating with D from *PtoNERD* (N72, N80, N81, and N135) (**Figure 2C**). These observations further demonstrate the important functions of L3 and L20 in *NERDL* and the importance of the SNP pairs under the epistasis model for phenotypic variation, and they help confirm the notion that the interaction of NERDL with *PtoNERD* contributes to wood formation. In addition, 3 SNPs (N11, N12, and N20) out of 11 also showed significant effects in the additive and dominant models, while the remaining epistatic effects involved pairs of SNPs that showed deficiency for both additive and dominant effects. These findings are consistent with the suggestion by Xu and Jia (2007) that epistatic variance affects genetic variance. Moreover, whether or not two SNP loci have additive and dominant effects does not influence whether they have epistatic effects. Interestingly, we detected an intragenic SNP pair, N4-N131, in which N4 had no effect on HEMC, whereas it did have pairwise effects (4.03%) on HEMC in combination with N131 (Supplementary Table S3). Epistasis analysis indicated that the lncRNA NERDL might interact with *PtoNERD* via similar pathways and that their complex interactions play a role in regulating tree growth and determining wood properties.

Our RT-qPCR analysis uncovered a positive correlation in expression of NERDL and its putative target (*r* = 0.62; **Figure 1C**). This finding is consistent with the results of association studies of additive and dominance effects, strongly suggesting that NERDL and its potential target affect phenotypic variation in a similar manner. NERDL and *PtoNERD* were more highly expressed in secondary tissues (xylem) than in primary tissues (cambium), indicating that they may be involved in secondary wood formation. The high abundance of the two transcripts in phloem and xylem and the 35 associations with growth and wood formation traits suggest that NERDL and *PtoNERD* together perform a regulatory function in secondary xylem formation in trees. The overlapping SNP L23/N3 also had significant effects on the transcript levels of NERDL and *PtoNERD* (**Figure 2B**). Analysis of genotype data indicated that the 15 haplotypes explained 1.06–13.16% of phenotypic variation, which was confirmed by the finding that significant SNPs are located in blocks of haplotypes associated with the same traits.

## Conclusion

In the present study, we identified *PtoNERD*, as an important player in the non-canonical RdDM pathway, as well as the lncRNA NERDL, which is located in the antisense strand and overlaps with the promoter region of *PtoNERD*. We used association (SNP-based and haplotype-based) studies and expression profiling to examine the common roles of NERDL and its putative target *PtoNERD* in tree growth and wood formation. Epistasis modeling helped reveal interactions between NERDL and *PtoNERD* for the traits D and FW, suggesting that the genetic interactions of NERDL and *PtoNERD* affect wood formation. Taken together, these results show that our method was successfully used to help decipher the allelic variation and genetic interactions of lncRNA and its target in trees through population genetics studies. The phenotype-related SNPs detected in this study could be applied to marker-assisted breeding in the near future.

## Data Archiving Statement

Sequence data for *NERDL* and *PtoNERD* in this article have been deposited in the GenBank Data Library (accession numbers KX344444-KX344531), and the transcriptome sequencing data are available in the SRA database (accession number SRP073689).

## Author Contributions

DZ conceived and designed experiment; WS and MQ performed the experiments and data analysis; WS wrote the manuscript; MQ and QD provided valuable advice on the manuscript; QD and DZ revised the manuscript; DZ obtained funding and is responsible for this article. All authors read and approved the manuscript.

## Conflict of Interest Statement

The authors declare that the research was conducted in the absence of any commercial or financial relationships that could be construed as a potential conflict of interest.

## Footnotes

1 http://www.ncbi.nlm.nih.gov

2 http://prosite.expasy.org/prosite.html

3 http://smart.embl-heidelberg.de//

4 http://www.maizegenetics.net/

5 http://pritch.bsd.uchicago.edu/structure.html

## Supplementary Material

FIGURE S1 The expression levels of seven genes involved in the NERD pathway in three tissues revealed by RNA-seq. The expression level of each gene was generated from the FPKM value and used to visualize the expression patterns of the genes according to the RNA-seq data.

FIGURE S2 Linkage disequilibrium within *NERDL* **(A)** and *PtoNERD* **(B)**. Pairwise correlations between SNPs were plotted against the physical distance between them (in base pairs). The curves indicate the non-linear regressions of *r*^2^ onto the physical distance (in base pairs).

FIGURE S3 The expression levels of NERDL and *PtoNERD* in three vascular tissues revealed by RNA-seq. The expression level of each gene was generated from the FPKM value and used to visualize the expression patterns of the genes according to the RNA-seq data.
