# Supplementary figures and images for: The Interactions between the Long Non-coding RNA NERDL and Its Target Gene Affect Wood Formation in Populus tomentosa
Source: Front Plant Sci. 2017 Jun 15;8:1035. doi: 10.3389/fpls.2017.01035 (PMC5475392; doi:10.3389/fpls.2017.01035)

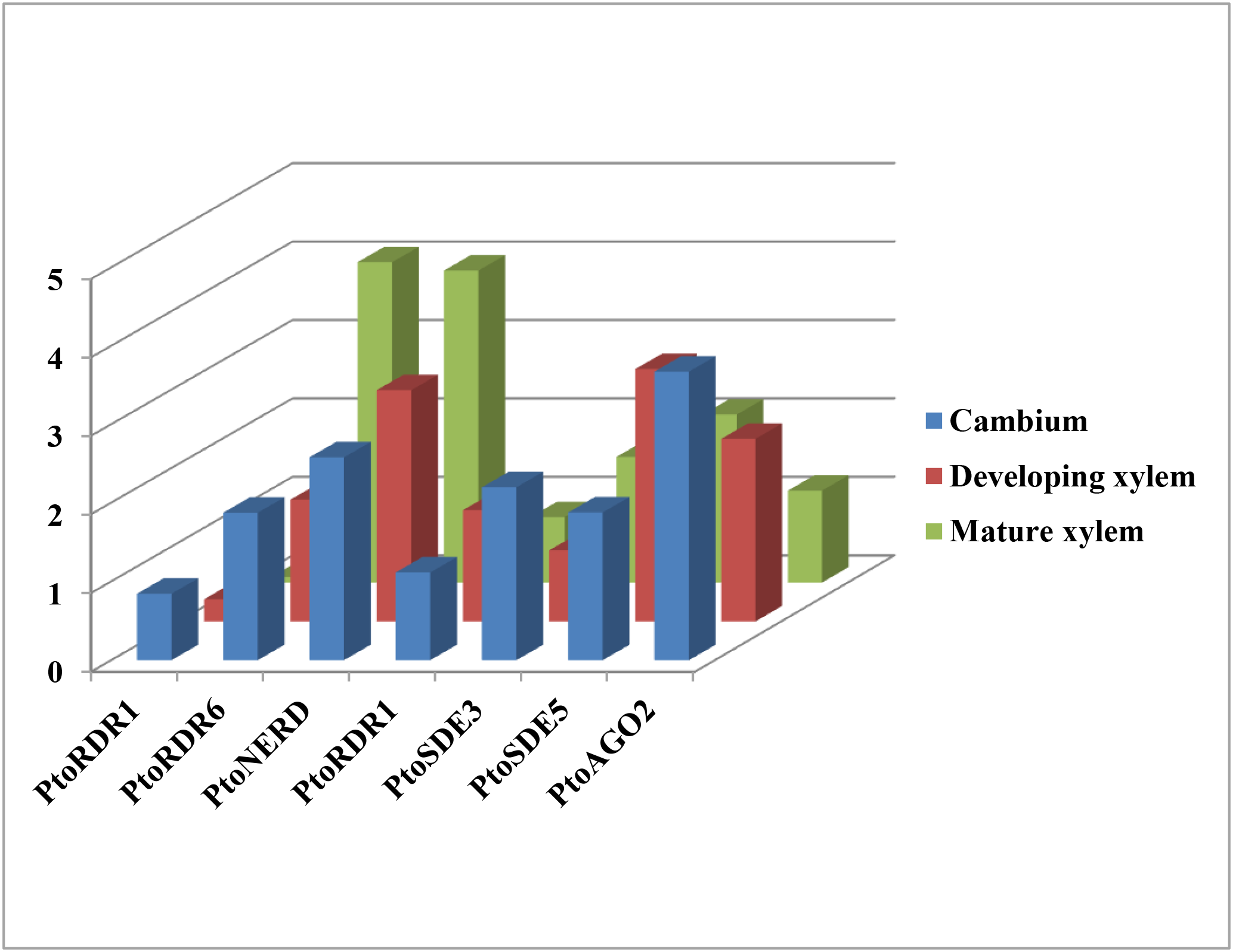

Supplement: FIGURE S1 — The expression levels of seven genes involved in the NERD pathway in three tissues revealed by RNA-seq. The expression level of each gene was generated from the FPKM value and used to visualize the expression patterns of the genes according to the RNA-seq data. [file Image_1.TIF]

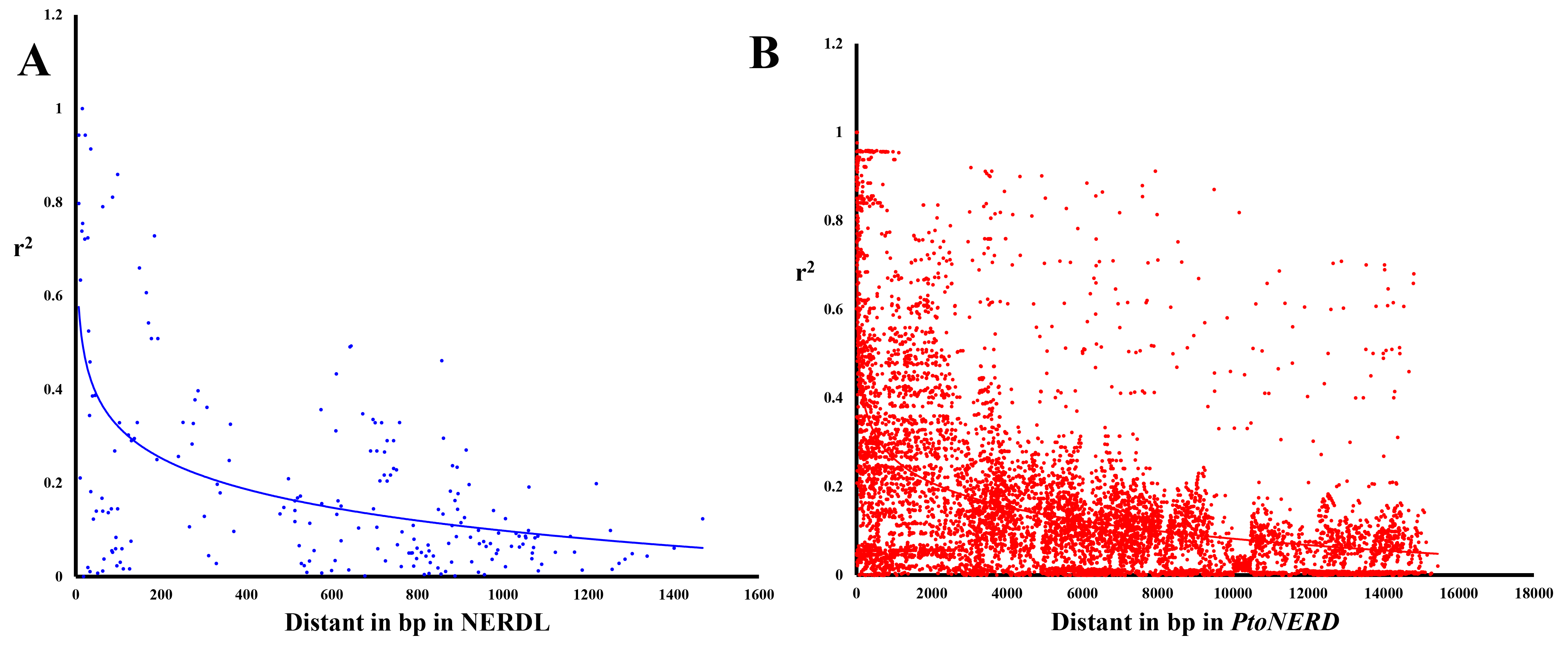

Supplement: FIGURE S2 — Linkage disequilibrium within NERDL (A) and PtoNERD (B). Pairwise correlations between SNPs were plotted against the physical distance between them (in base pairs). The curves indicate the non-linear regressions of r2 onto the physical distance (in base pairs). [file Image_2.TIF]

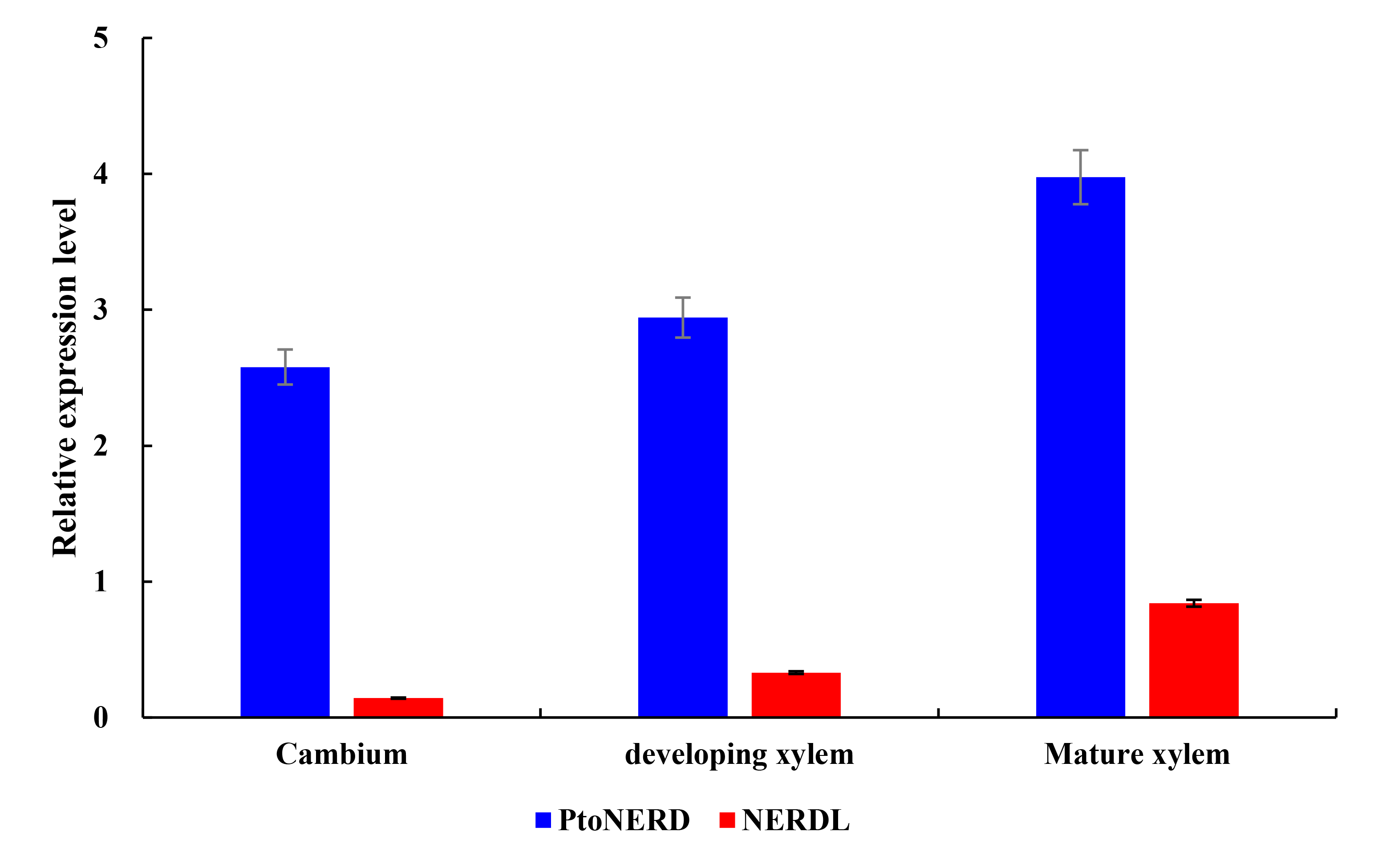

Supplement: FIGURE S3 — The expression levels of NERDL and PtoNERD in three vascular tissues revealed by RNA-seq. The expression level of each gene was generated from the FPKM value and used to visualize the expression patterns of the genes according to the RNA-seq data. [file Image_3.TIF]
