# Supplementary material for: The Interactions between the Long Non-coding RNA NERDL and Its Target Gene Affect Wood Formation in Populus tomentosa
Source: Front Plant Sci. 2017 Jun 15;8:1035. doi: 10.3389/fpls.2017.01035 (PMC5475392; doi:10.3389/fpls.2017.01035)
Supplement: Supplementary file 4 [file Table_1.DOC]

| **Gene** | **Forward primer(5′→3′ )** | **Reverse primer (5′→3′ )** |
| --- | --- | --- |
| NERDL | AATCGCTCGAGATTTTGCAAA | TTTGCAGTCGAGTGGTTAGATCA |
| *PtoNERD* | AATCTGGCTTCTATGCCTTCACA | ACAGAGGCTGGTGCATTGC |
| *Actin* | TTCATTTCACATCTTCCCCTTTT | GATCTCTGTGTGGGCGTCTGT |

**Table S1** The primers used for RT-qPCR in this study.
