# Supplementary material for: The Interactions between the Long Non-coding RNA NERDL and Its Target Gene Affect Wood Formation in Populus tomentosa
Source: Front Plant Sci. 2017 Jun 15;8:1035. doi: 10.3389/fpls.2017.01035 (PMC5475392; doi:10.3389/fpls.2017.01035)
Supplement: Supplementary file 5 [file Table_2.DOC]

***Table S2*** *Signiﬁcant SNPs associated with all traits calculated by MLM and the additive effect, dominant effect, and r^2^ (phenotypic contributions) of the SNPs from NERDL and PtoNERD associated with each trait in the association population of Populus tomentosa.*

| Position | Associated traits | SNPs locus | Position | P-value | Q-value | Additive effect(%) | Dominant effect(%) | R^2^ (%) |
| --- | --- | --- | --- | --- | --- | --- | --- | --- |
| NERDL |  |  |  |  |  |  |  |  |
|  | AC (%) | SNP9 | - | 5.64E-04 | 6.78E-02 | 11.82 | 7.88 | 5.64 |
|  | D (cm) | SNP18 | - | 5.93E-04 | 6.85E-02 | 2.43 | -9.98 | 6.10 |
|  |  | L23/N3 | Promoter(NERD) | 2.69E-05 | 6.01E-02 | 6.45 | 3.65 | 4.70 |
|  | HEC (%) | SNP8 | - | 3.37E-04 | 6.03E-02 | 12.82 | -10.34 | 5.92 |
|  |  | SNP9 | - | 5.05E-04 | 6.53E-02 | 12.95 | -9.56 | 6.54 |
| *PtoNERD* |  |  |  |  |  |  |  |  |
|  | AC (%) | SNP12 | Promoter | 4.14E-04 | 7.57E-02 | 14.50 | 12.32 | 6.88 |
|  |  | SNP103 | EXON11 | 8.60E-04 | 8.13E-02 | 7.85 | 6.80 | 6.10 |
|  | D (cm) | L23/N3 | Promoter | 2.69E-05 | 6.01E-02 | 6.45 | 3.65 | 4.70 |
|  |  | SNP30 | EXON1 | 5.36E-04 | 7.82E-02 | 0.80 | -1.82 | 4.21 |
|  |  | SNP31 | EXON1 | 5.52E-04 | 7.83E-02 | 3.56 | 0.90 | 5.03 |
|  |  | SNP50 | INTRON3 | 6.32E-04 | 7.87E-02 | 6.09 | 7.42 | 4.69 |
|  |  | SNP54 | INTRON3 | 6.64E-04 | 7.88E-02 | 0.86 | 0.55 | 4.39 |
|  |  | SNP73 | INTRON4 | 7.35E-04 | 7.89E-02 | 3.51 | 2.37 | 4.33 |
|  |  | SNP131 | 3'UTR | 9.88E-04 | 8.15E-02 | 5.71 | 3.84 | 5.50 |
|  | FW (µm) | SNP9 | Promoter | 1.36E-04 | 7.13E-02 | 1.06 | 2.42 | 3.90 |
|  |  | SNP11 | Promoter | 2.32E-04 | 7.35E-02 | 0.52 | 0.61 | 7.46 |
|  |  | SNP12 | Promoter | 3.36E-04 | 7.45E-02 | 2.80 | -1.44 | 8.57 |
|  |  | SNP15 | Promoter | 4.37E-04 | 7.69E-02 | 1.13 | 2.74 | 5.67 |
|  |  | SNP16 | Promoter | 4.93E-04 | 7.71E-02 | 1.15 | 2.47 | 5.38 |
|  |  | SNP18 | Promoter | 4.99E-04 | 7.75E-02 | 0.54 | -0.03 | 4.52 |
|  |  | SNP19 | Promoter | 5.05E-04 | 7.80E-02 | 1.30 | 2.24 | 4.52 |
|  |  | SNP20 | Promoter | 5.19E-04 | 7.81E-02 | 0.65 | 1.47 | 6.14 |
|  |  | SNP21 | Promoter | 5.29E-04 | 7.81E-02 | 0.55 | 0.62 | 6.11 |
|  |  | SNP33^ns^ | EXON1 | 5.59E-04 | 7.85E-02 | 1.45 | -1.45 | 4.13 |
|  |  | SNP49 ns | EXON2 | 5.78E-04 | 7.86E-02 | 0.22 | 0.26 | 4.58 |
|  |  | SNP75 ^ns^ | EXON6 | 7.41E-04 | 7.89E-02 | 3.42 | -2.30 | 7.67 |
|  |  | SNP78 | INTRON6 | 7.53E-04 | 7.90E-02 | 0.42 | 2.45 | 7.35 |
|  |  | SNP105 | EXON11 | 9.65E-04 | 8.14E-02 | 1.07 | 2.37 | 6.24 |
|  | HEC (%) | SNP5 | Promoter | 5.02E-05 | 6.41E-02 | 9.33 | -5.40 | 4.10 |
|  |  | SNP8 | Promoter | 1.15E-04 | 6.92E-02 | 6.16 | 9.37 | 4.46 |
|  |  | SNP9 | Promoter | 1.20E-04 | 7.03E-02 | 6.26 | 8.00 | 4.74 |
|  |  | SNP10 | Promoter | 2.10E-04 | 7.27E-02 | 5.28 | 11.77 | 5.99 |
|  |  | SNP11 | Promoter | 3.27E-04 | 7.37E-02 | 2.10 | 3.28 | 4.98 |
|  |  | SNP105 | EXON11 | 9.73E-04 | 8.15E-02 | 5.18 | 10.99 | 5.96 |
|  | LC (%) | SNP93 | INTRON8 | 7.85E-04 | 8.25E-02 | 0.17 | -0.05 | 4.46 |
