# Supplementary material for: The Interactions between the Long Non-coding RNA NERDL and Its Target Gene Affect Wood Formation in Populus tomentosa
Source: Front Plant Sci. 2017 Jun 15;8:1035. doi: 10.3389/fpls.2017.01035 (PMC5475392; doi:10.3389/fpls.2017.01035)
Supplement: Supplementary file 6 [file Table_3.DOC]

***Table S3*** *Associations detected by MDR 3.0.2 under the epistasis gene model.*

| Associated traits | Attribute A | Single effect (% A) | Attribute B | Single effect (% B) | Effect of interaction (%) | Information gain (%) |
| --- | --- | --- | --- | --- | --- | --- |
| D(cm) |  |  |  |  |  |  |
|  | N80 | 1.45 | N72 | 1.19 | 1.45 | -1.19 |
|  | N81 | 1.14 | N72 | 1.19 | 1.45 | -0.88 |
|  | N81 | 1.14 | N80 | 1.45 | 1.45 | -1.14 |
|  | N135 | 12.28 | N72 | 1.19 | 4.08 | -9.39 |
|  | N135 | 12.28 | N80 | 1.45 | 5.81 | -7.92 |
|  | N135 | 12.28 | N81 | 1.14 | 2.77 | -10.65 |
|  | L20 | 3.79 | N72 | 1.19 | 7.14 | 2.16 |
|  | L20 | 3.79 | N80 | 1.45 | 3.48 | -1.77 |
|  | L20 | 3.79 | N81 | 1.14 | 2.12 | -2.81 |
|  | L20 | 3.79 | N135 | 12.28 | 8.52 | -7.55 |
| FW (µm) |  |  |  |  |  |  |
|  | N12 | 4.59 | N11 | 2.48 | 2.11 | -4.95 |
|  | N20 | 2.13 | N11 | 2.48 | 7.94 | 3.33 |
|  | N20 | 2.13 | N12 | 4.59 | 10.37 | 3.65 |
|  | N98 | 0.28 | N11 | 2.48 | 2.48 | -0.28 |
|  | N98 | 0.28 | N12 | 4.59 | 2.96 | -1.91 |
|  | N98 | 0.28 | N20 | 2.13 | 2.77 | 0.35 |
|  | N135 | 0.69 | N11 | 2.48 | 2.27 | -0.9 |
|  | N135 | 0.69 | N12 | 4.59 | 2.77 | -2.51 |
|  | N135 | 0.69 | N20 | 2.13 | 2.62 | -0.21 |
|  | N135 | 0.69 | N98 | 0.28 | 0.27 | -0.71 |
|  | L3 | 0.11 | N11 | 2.48 | 1.53 | -1.05 |
|  | L3 | 0.11 | N12 | 4.59 | 2.96 | -1.73 |
|  | L3 | 0.11 | N20 | 2.13 | 2.77 | 0.53 |
|  | L3 | 0.11 | N98 | 0.28 | 0.97 | 0.58 |
|  | L3 | 0.11 | N135 | 0.69 | 1.39 | 0.59 |
| AC (%) |  |  |  |  |  |  |
|  | N2 | 1.42 | N1 | 1.2 | 3.48 | 0.86 |
|  | N63 | 4.81 | N1 | 1.2 | 1.26 | -4.74 |
|  | N63 | 4.81 | N2 | 1.42 | 1.89 | -4.34 |
|  | N120 | 2.81 | N1 | 1.2 | 0.24 | -3.77 |
|  | N120 | 2.81 | N2 | 1.42 | 1.37 | -2.86 |
|  | N120 | 2.81 | N63 | 4.81 | 2.27 | -5.34 |
| Angle (°) |  |  |  |  |  |  |
|  | N52 | 4.18 | N49 | 0.08 | 1.75 | -2.51 |
|  | N64 | 6.03 | N49 | 0.08 | 0.62 | -5.49 |
|  | N64 | 6.03 | N52 | 4.18 | 4.84 | -5.37 |
|  | N82 | 1.24 | N49 | 0.08 | 1.24 | -0.08 |
|  | N82 | 1.24 | N52 | 4.18 | 2.81 | -2.6 |
|  | N82 | 1.24 | N64 | 6.03 | 1.24 | -6.03 |
|  | N90 | 3.64 | N49 | 0.08 | 7.39 | 3.67 |
|  | N90 | 3.64 | N52 | 4.18 | 2.77 | -5.05 |
|  | N90 | 3.64 | N64 | 6.03 | 4.89 | -4.78 |
|  | N90 | 3.64 | N82 | 1.24 | 1.89 | -2.98 |
| FL(mm) |  |  |  |  |  |  |
|  | N53 | 1.46 | N31 | 3.55 | 4.68 | -0.33 |
|  | N63 | 4.81 | N31 | 3.55 | 3.46 | -4.9 |
|  | N63 | 4.81 | N53 | 1.46 | 0.4 | -5.87 |
|  | N77 | 2.3 | N31 | 3.55 | 2.19 | -3.65 |
|  | N77 | 2.3 | N53 | 1.46 | 0.08 | -3.68 |
|  | N77 | 2.3 | N63 | 4.81 | 8.97 | 1.87 |
|  | N109 | 1.22 | N31 | 3.55 | 0.56 | -4.2 |
|  | N109 | 1.22 | N53 | 1.46 | 0 | -2.68 |
|  | N109 | 1.22 | N63 | 4.81 | 3.69 | -2.34 |
|  | N109 | 1.22 | N77 | 2.3 | 0.56 | -2.95 |
| H (m) |  |  |  |  |  |  |
|  | N84 | 0.28 | N52 | 6.81 | 2.6 | -4.49 |
|  | N85 | 1.93 | N52 | 6.81 | 5.51 | -3.23 |
|  | N85 | 1.93 | N84 | 0.28 | 2.3 | 0.09 |
|  | N108 | 0.28 | N52 | 6.81 | 6.62 | -0.47 |
|  | N108 | 0.28 | N84 | 0.28 | 0.16 | -0.4 |
|  | N108 | 0.28 | N85 | 1.93 | 1.14 | -1.07 |
| HEC (%) |  |  |  |  |  |  |
|  | N60 | 1.75 | N50 | 1.24 | 2.3 | -0.69 |
|  | N61 | 0.3 | N50 | 1.24 | 1.14 | -0.41 |
|  | N61 | 0.3 | N60 | 1.75 | 0.68 | -1.37 |
|  | N119 | 4.68 | N50 | 1.24 | 4.68 | -1.25 |
|  | N119 | 4.68 | N60 | 1.75 | 1.89 | -4.53 |
|  | N119 | 4.68 | N61 | 0.3 | 1.31 | -3.68 |
| HEMC (%) |  |  |  |  |  |  |
|  | N64 | 0.56 | N4 | 0 | 0 | -0.56 |
|  | N69 | 1.58 | N4 | 0 | 1.58 | -2.32 |
|  | N69 | 1.58 | N64 | 0.56 | 0.66 | -1.48 |
|  | N131 | 3.6 | N4 | 0 | 4.03 | 0.43 |
|  | N131 | 3.6 | N64 | 0.56 | 3.33 | -0.84 |
|  | N131 | 3.6 | N69 | 1.58 | 2.3 | -2.88 |
| LC (%) |  |  |  |  |  |  |
|  | N18 | 0.21 | N3 | 3.97 | 1.05 | -3.13 |
|  | N69 | 0.14 | N3 | 3.97 | 0.85 | -3.26 |
|  | N69 | 0.14 | N18 | 0.21 | 1.14 | 0.79 |
|  | N131 | 1.59 | N3 | 3.97 | 1.68 | -3.88 |
|  | N131 | 1.59 | N18 | 0.21 | 0.48 | -1.32 |
|  | N131 | 1.59 | N69 | 0.14 | 2.3 | 0.57 |
| V (m^3^) |  |  |  |  |  |  |
|  | N38 | 5.59 | N21 | 1.86 | 2.25 | -5.19 |
|  | N64 | 7.15 | N21 | 1.86 | 8.41 | -0.6 |
|  | N64 | 7.15 | N38 | 5.59 | 5.52 | -7.21 |
|  | N72 | 1.19 | N21 | 1.86 | 2.6 | -0.45 |
|  | N72 | 1.19 | N38 | 5.59 | 0.77 | -6 |
|  | N72 | 1.19 | N64 | 7.15 | 8.41 | 0.07 |
|  | N83 | 2.05 | N21 | 1.86 | 4.91 | 1 |
|  | N83 | 2.05 | N38 | 5.59 | 5.9 | -1.74 |
|  | N83 | 2.05 | N64 | 1.19 | 4.03 | -5.17 |
|  | N83 | 2.05 | N72 | 7.15 | 0.66 | -2.58 |
|  | N97 | 1.98 | N21 | 1.86 | 2.88 | -0.96 |
|  | N97 | 1.98 | N38 | 5.59 | 3.63 | -3.93 |
|  | N97 | 1.98 | N64 | 7.15 | 2.42 | -6.72 |
|  | N97 | 1.98 | N72 | 1.19 | 1.98 | -1.19 |
|  | N97 | 1.98 | N83 | 2.05 | 1.98 | -2.05 |
